# Association of endometriosis with interstitial cystitis in chronic pelvic pain syndrome: Short narrative on prevalence, diagnostic limitations, and clinical implications

**DOI:** 10.5339/qmj.2021.50

**Published:** 2021-10-07

**Authors:** Tariq F. Al-Shaiji, Dalal H. Alshammaa, Mariam M. Al-Mansouri, Abdullatif E. Al-Terki

**Affiliations:** ^1^Urology Unit, Department of Surgery, Amiri Hospital, Kuwait City, Kuwait E-mail: tshaiji@gmail.com; ^2^Department of Obstetrics and Gynecology, Maternity Hospital, Kuwait City, Kuwait; ^3^Department of Obstetrics and Gynecology, Maternity Hospital, Kuwait City, Kuwait; ^4^Urology Unit, Department of Surgery, Amiri Hospital, Kuwait City, Kuwait

**Keywords:** endometriosis, interstitial cystitis, chronic pelvic pain syndrome, evil twins’ syndrome

## Abstract

Introduction: Chronic pelvic pain (CPP) is a diagnostic and therapeutic challenge affecting women of all ages globally. The syndrome is not well understood, but the association of interstitial cystitis (IC) with endometriosis in causing CPP should not be overlooked in managing this cohort. Herein, we present a mini review of this association to evaluate the literature in determining the prevalence of endometriosis and IC concomitantly in patients with CPP, diagnostic limitations, and clinical implications.

Methods: A Medline search of the key words “evil twins’ syndrome,” “interstitial cystitis,” “bladder pain syndrome,” and “endometriosis” was conducted for full-text articles published in English over the past 20 years. The search yielded 40 articles, of which 21 were selected. Cross-referencing bibliographies of each publication yielded an additional 25 references.

Results: Both endometriosis and IC share a similar array of symptoms that are often exacerbated during the perimenstrual period. Multiple authors have reported the frequent coexistence of these two conditions. Over 80% of patients with CPP were found to have both conditions. The prevalence of endometriosis and IC coexistence was greater than that of each condition separately.

Conclusions: It is crucial to look beyond the traditionally diagnosed endometriosis as the cause of CPP. This is true especially in patients whose previous treatment was ineffective. Simultaneous assessment for both conditions is essential to avoid the frequently delayed diagnosis and prevent unsuccessful medical and surgical therapies.

## Introduction

Chronic pelvic pain (CPP) is a syndrome with diagnostic and therapeutic challenge that affects women of all ages globally. A standardized definition or diagnostic method for CPP has not been established. It is often defined as noncyclic lower abdominal or pelvic pain that lasts at least 6 months and may be intermittent or constant and may be exacerbated by menstruation or intercourse. Pain severity can range from mild to debilitating pain. It often affects the patients’ quality of life. Investigation results are often vague, and the cause of the pain may not be identified. Given the paucity in epidemiological data, the true incidence of CPP is unknown. However, it is estimated to affect 1 in 7 women and 9 million women in the United States^1^. A systematic review conducted by the World Health Organization (WHO) in 2006 estimated the prevalence of CPP to range from 4% to 43.4% globally (Table 1)^2^. The diagnosis of CPP poses a clinical challenge because of an extensive list of nonspecific symptoms, an equally extensive list of possible diagnoses, and the need for invasive testing. The differential diagnoses of CPP encompass multiple specialties, including gastrointestinal, gynecologic, urologic, musculoskeletal, and psychiatric fields. Moreover, < 20% of patients with CPP seek medical treatment^3^. CPP accounts for 10%–15% of gynecological referrals, 10%–12% of hysterectomies, and 40% of gynecologic laparoscopic procedures^3,4^. During laparoscopy, pathology is not found in 35% of the cases, and when pathology is identified, it is often unclear whether it is the true cause of the CPP, which further adds to the diagnostic challenge^5^. CPP has a debilitating effect on daily activities and quality of life in 50%–60% of women^6^. It is linked to multiple conditions, including depression which is prevalent in up to 50% of women with CPP and anxiety^2,4,6^. Patients with CPP are often treated empirically for a presumed diagnosis with poor response and high recurrence rates. With their frustration of the treatment outcomes, these patients seek care from different physicians and undergo multiple unsuccessful medical and surgical therapies. Medication ingestion in women with CPP is three times higher than that of healthy women, while gynecological surgical procedures are four times higher^6^. CPP poses a massive financial healthcare burden. The estimated annual outpatient health care cost spent in the United States for the treatment of CPP is $881.5 million^2^. A cross-sectional study of inpatient care of CPP estimated that the yearly expenditure reached $25 million^7^. Moreover, 92.9% of inpatients underwent surgical intervention, and the most common were hysterectomy (47.1%) and laparoscopy (25.8%)^7^.

Traditionally, endometriosis was considered the most common cause of CPP. Recently, interstitial cystitis (IC) has been emerging as a major player in the CPP complex. Numerous authors have reported the coexistence of these two conditions and the predicament faced in attributing which of the two caused CPP when both conditions are present. In 2002, Chung et al. retrospectively reviewed 60 patients with CPP and found a high likelihood of the two conditions co-occurring, coining the term the “evil twins” of CPP^3^. Although this term never gained standardization, the co-occurrence of these two conditions garnered much attention. Thus, this review aimed to determine the prevalence of endometriosis and IC found concomitantly in patients with CPP and to compare the prevalence of endometriosis and IC individually.

Bladder-origin pelvic pain has become widely recognized since the introduction IC and painful bladder syndrome (PBS) in 1887 and 1957, respectively^8,9^. Over time, as the definition of IC evolved, so has its reported prevalence^10^. According to the European Society for the Study of Interstitial Cystitis definition, PBS was diagnosed in cases where CPP and at least one urinary symptom were experienced. IC was defined as CPP with at least one urinary symptom and the presence of glomerulations in at least two quadrants^10^. In the United States, approximately 1 in 4.5 women may have IC^5^. In addition, 85% of patients with CPP have IC, and a positive potassium sensitivity test (PST) was found in 82% of cases with CPP^5^. IC presents with a heterogeneous array of symptoms including urinary urgency, frequency, and nocturia. It is also associated with sexual dysfunction with deep dyspareunia, bladder pain during and after intercourse, and the urge to urinate during intercourse. Pain or lack of sensation in the genital area as well as reduced libido, sexual arousal, and orgasm frequency have also been reported^11^. Furthermore, 75% of patients with IC report exacerbation of symptoms following sexual intercourse^12^. Different studies have reported that the rate of sexual dysfunction in patients with IC range from 13% to 87%^11,13^. Early, mild disease presents with intermittent symptoms, which may progress to persistent debilitating disease. Early recognition is challenging as IC shares presenting symptoms with multiple other conditions, making it difficult to distinguish IC from conditions such as endometriosis, urinary tract infections, vulvodynia, and overactive bladder (OAB)^14^. The diagnosis of IC is generally delayed if the patient has had experienced symptoms for several years and undergone multiple pelvic surgeries^12,13^. IC is usually symptomatic for 4–7 years before the diagnosis is made^12,14^. Most patients are diagnosed in their early 40s, 30% are diagnosed in their 30s, and 27% are diagnosed within the ages of 19–34^13^.

Endometriosis is believed to be one of the most common causes of CPP accounting for 71%–87% of the cases^3,8,14,15^. Endometriosis affects 5%–15% of women of reproductive age^3,5,12^. The diagnosis of endometriosis has increased in the past 15–20 years. Pain is experienced by 50%–70% of women with endometriosis^16^. In addition to pain, endometriosis causes dysmenorrhea, vulvodynia, dyspareunia, dyschezia, menorrhagia, and infertility. Dyspareunia is reported by 86% of patients, 69% avoid intercourse, and 19% are no longer sexually active^17^. The diagnosis can only be confirmed by laparoscopy and histology, which are expensive and invasive and require general anesthesia. As a result, the diagnosis is often delayed up to 12 years^16^. Despite laparoscopy being the accepted gold standard for the diagnosis of endometriosis, many national and international guidelines have recommended initiation of empiric therapy and monitoring for response. Therefore, many women will be diagnosed clinically without histologic confirmation^18^. Only 28%–35% of patients with CPP have biopsy-confirmed endometriosis^5^. Visual identification of lesions is not sufficient in confirming the diagnosis^19^. Treatment in many patients presumed to suffer from endometriosis produces temporary, insufficient pain relief in a progressive disease with high recurrence rates reaching 50%^3^. These patients were more likely to undergo multiple surgical procedures, use two or more medications to treat endometriosis, undergo appendectomies and cholecystectomies, and develop mood disorders^20^. Endometriosis is associated with multiple comorbid pain conditions, including irritable bowel syndrome, fibromyalgia, chronic headache, depression, and anxiety. In a previous study 34, thirty sex percent of patients with endometriosis reported that their quality of life and academic performance were affected^20^.

## Methods

A Medline search of “interstitial cystitis” OR “bladder pain syndrome” OR “evil twins’ syndrome” AND “endometriosis” was conducted. The search was limited to English full-text articles that were published over the past 20 years, with female patients aged 13–44 years. The inclusion criteria were limited to this age group, as the majority of cases of endometriosis and IC initially present and are investigated during this age. Although the diagnosis of both conditions is often delayed with years of symptoms before diagnosis, the majority of patients are diagnosed in their early 40s, 30% are diagnosed in their 30s, and 27% are diagnosed within the ages of 19–34 years^13^. Case reports, clinical trials, meta-analyses, observational studies, and reviews were included. The search yielded 40 results. Cross-referencing bibliographies of each publication yielded an additional 25 references. As this was a review of published literature, institutional ethical board approval was not required.

## Results

The association of IC with endometriosis has been well reported by multiple authors, and their findings are summarized in Table 2. Chung et al. found that 89% of patients with CPP who were presumed to have endometriosis had a positive PST, suggesting that IC rather than endometriosis may be the source of CPP^5^. Of all patients with IC, 77.5% had urinary symptoms, while 76.5% of the endometriosis group had urinary symptoms^3^. In the IC group, 81% had histologically confirmed endometriosis^3^. In a prospective analysis of 178 patients with CPP undergoing laparoscopy and cystoscopy with bladder hydrodistension, Chung et al. found that 65% of patients have both conditions, 11% had endometriosis without evidence of IC, and 25% had IC alone^15^. Among patients with endometriosis, 86% were also diagnosed with IC, and 72% of patients with IC were also diagnosed with endometriosis^15^. The study results demonstrated that the two diagnoses much more commonly exist concurrently than for each of them to be diagnosed individually. The study also deduced that if cystoscopy had only been performed in patients with urinary symptoms, 20% of the patients with IC would have been missed^15^. The relationship between IC and endometriosis was further confirmed by Cheng et al. who recruited 150 women complaining of CPP. They were investigated with cystoscopy and hydrodistension as well as laparoscopy: 53% were found to have PBS, 60% with PBS also had endometriosis, 32% of the patients had IC diagnosed by some degree of glomerulations on cystoscopy, and 18% of the patients had both bladder glomerulations and endometriosis. Of the 90 women with endometriosis, 27 (30%) had IC and 45 (50%) had PBS^10^. This study, unlike its predecessors, investigated both IC and PBS.

Paulson et al. investigated concurrence of endometriosis and IC in 66% of patients. The study population included 162 women complaining of CPP both with and without urinary symptoms. In patients who were diagnosed with IC through cystoscopy with hydrodistension, 72% had urinary symptoms^21^. Before the study, 76 (47%) patients were receiving ovulation suppression using oral contraceptive pills to treat their CPP, and the vast majority of the patients (68%) reported no improvement in their pain. Of these 76 patients, 53 (71%) had both endometriosis and IC^21^. The mean Pelvic Pain and Urgency/Frequency Patient Symptom scale (PUF) scores for the group with both diagnoses was similar to that of the group with IC alone. The PUF score of the group with isolated endometriosis was significantly lower, suggesting the pain to be largely of bladder origin^21^.

The coexistence of IC and endometriosis was less common among adolescent and young-adult patients with CPP. In a case series, Rackow et al. evaluated the cause of CPP in 28 young women aged 13–25 years. Simultaneous laparoscopy and cystoscopy were performed; 25% of the patients had evidence of coexisting endometriosis and IC, 14% had isolated IC, and 39% had endometriosis alone^13^. Of the participants who were sexually active, 48% reported dyspareunia, and 71% who were diagnosed with both IC and endometriosis had complained of dyspareunia. In comparison, dyspareunia was experienced in 75% of participants with IC only and 38% of participants with endometriosis only^13^. Preoperative symptoms were not found to be reliable in predicting the diagnosis. Therefore, simultaneous evaluation of both the pelvis and bladder was recommended^13^. Smorgick et al. examined a similarly young population. The rate of IC and endometriosis coexistence was 16%, which was lower than the rate of 65% in the adult population^20^. This may be explained by the younger age group and early diagnosis and hence shorter follow-up. The study population underwent their first surgery at a median of 24 months from the onset of symptoms, while surgery and diagnosis in older adults are usually delayed at an average of 8–11.7 years^20^. The study population also had a strong family history and awareness of endometriosis. Given their young age, a surgically confirmed diagnosis may have increased parental comfort in initiating hormonal therapy^20^.

Endometriosis commonly recurs after surgery with an annual recurrence rate of 0.9% in the first year. Recurrence rate reaches 13.6% at the eighth postoperative year^19^. In cases where symptoms recur rapidly, recurrence of endometriosis is improbable, and other coexisting causes of pain, mainly IC, need to be considered^19^. Up to 25%–40% of patients who undergo hysterectomy as a treatment of CPP will continue to have pain postoperatively^21,22^. In a previous study, Chung MK investigated a population of 111 women with persistent CPP post-hysterectomy using the PUF scale and PST, and IC was found in 79% of the patients. These patients were then treated for IC and experienced marked improvements in their PUF scores of 34.2%^22^. IC is commonly diagnosed in women who had undergone hysterectomy or pelvic surgery. Patients frequently report urinary symptoms that commence after surgery. In a case–control study by Langenberg et al., patients with IC were asked to identify the index date of initial urinary symptoms as well as any previous surgical procedures during their lifetime, previous 12 months, and previous month. Patients with IC/PBS had a higher history of having undergone surgical procedures than the controls^23^. IC cases were found to have higher incidence of CPP, suggesting that the indication for surgery rather than surgery itself was the risk factor of IC. CPP as the indication for surgery had the highest association odds ratio (OR) of 4.9 (confidence interval 3.1–7.9)^23^. To distinguish whether the cause of CPP was actually undiagnosed IC, Langenberg et al. identified the date of initial urinary symptoms and any similar symptoms in the previous 5 years in all patients with CPP. In 73% of the cases, CPP preceded urinary symptoms by >12 months^23^. This study, however, did not identify the indication for previous surgeries, histological findings, and intraoperative findings and hence whether endometriosis or other pelvic pathology was found^23^. Antecedent CPP is found in 38% of IC cases^24^. The risk of IC increased with antecedent CPP with an OR of 4.5^24^. Warren et al. reported that patients with IC had higher rates of antecedent visualized or histologically confirmed endometriosis than controls^25^. In a case–control study by Ingber et al., women with IC were found to have high rates of having undergone laparoscopic pelvic surgery and hysterectomy when compared with controls, and 42.3% of cases had hysterectomies in comparison with 21.4% of controls^26^. Endometriosis was also more commonly diagnosed in the IC group (25.6%) compared with 9.8% in the control group, and 68% of hysterectomies were performed before the diagnosis of IC^26^. IC was diagnosed 1–5 years after hysterectomy^26^. Only 21% of hysterectomies are performed following the diagnosis of IC, suggesting that previous hysterectomies may have been performed to relieve CPP caused by undiagnosed IC^26^.

## Discussion

### Similar symptomatology

IC and endometriosis share the same pathogenesis, with mast cell infiltration and degranulation occurring in both conditions^5^. In addition, viscerovisceral hyperalgesia allows painful stimuli from the bladder to be transmitted to the spinal cord and perceived as pain anywhere in the pelvis. Thus, it is difficult to identify the organ that generates pain and whether the pain is caused by multiple or a single origin^5^. Endometriosis and IC also share symptomatology. Both conditions cause dyspareunia and vulvodynia and are associated with menstrual exacerbation of symptoms (Table 3). Premenstrual flares of IC were found in 75% of the patients^5^. Powell-Boone et al. recruited seven women with IC and eight and asked them to document and track their daily bladder and other body pains in a diary. In the IC group, pain scores and urinary frequency were the highest in the perimenstrual period. Cystometry performed during the follicular premenstrual period showed that bladder pain was evoked with lower intravesical infused volume and pressure when compared with the results obtained during the luteal period^27^. Dyspareunia and sexual dysfunction are also reported in both conditions. Dyspareunia is found in 40%–57% of cases of IC and 43%–62% of endometriosis^11,28^. Moreover, 60%–80% of patients with endometriosis undergoing surgery experience dyspareunia, while 50%–90% of patients on medical treatment suffer from dyspareunia^28^. In comparison, rates of sexual dysfunction in patients with IC ranged from 13% to 87% in different studies. The estimated rate of sexual dysfunction in the general population in the United States is 40%. Bogart et al. reported that two-thirds of patients with IC experienced sexual dysfunction^11^. In a prospective observational study, Yong et al. evaluated 150 women with dyspareunia who sought treatment for deep and superficial dyspareunia and associated factors using patient standardized questionnaires. In the group with concurrent deep and superficial dyspareunia, 18.2% of the patients had a previous diagnosis of endometriosis, 24.2% complained of bladder-related symptoms, and 9.1% had received a prior diagnosis of IC. These patients had higher reported levels of dyspareunia pain^18^. Owing to the discomfort of discussing sexual dysfunction, only 10%–20% of patients sought medical assistance^11^. Patients with CPP are often hesitant to report sexual dysfunction when seeking healthcare^28^.

Paulson et al. found high rates of anterior vaginal wall tenderness in patients with endometriosis and IC, and 61% of the patients had both IC and endometriosis. Patients with both concurrent diagnoses had high pain scores and rates of dysmenorrhea and dyspareunia when compared with patients with isolated IC and isolated endometriosis^4^. Dyspareunia is traditionally often attributed to endometriosis; however, some studies have reported higher rates of dyspareunia in patients with IC than in patients with endometriosis. Rackow et al. reported that dyspareunia was experienced in 75% of patients with IC alone and 38% of patients with endometriosis. In comparison, 71% of patients with both IC and endometriosis reported dyspareunia; a rate that was similar to those with IC alone^13^. The association of IC with vulvodynia is not well understood. The reported prevalence of vulvodynia ranged from 7% to 28%^14,29,30^. The prevalence of vulvodynia in patients with IC has ranged from 51.4% to 85.1%^14,30^. Among patients with persistent vulvodynia, 22% had IC^14^. In a study by Kahn et al. where 122 patients with vulvodynia underwent PUF and PST, 84% of women with vulvodynia had a positive PST and 80% of these patients reported urinary urgency and frequency^29^. The study suggested that vulvodynia was a symptom of a more complex disease, i.e., IC, originating in the bladder. A common etiology for the two conditions has been suggested as both the vulva and bladder originate from the urogenital sinus and share neurologic innervation by the common sacral nerve^14^. IC profoundly affects sexual function with 23.4% of patients reporting abstinence from sexual activity in the previous year compared with 9% of controls, and 50% of the women avoided intercourse^30^.

### Limitations and clinical implications

The true prevalence of both IC and biopsy-confirmed endometriosis is unknown because of several limitations**.** Laparoscopy is considered the gold standard for the diagnosis of endometriosis. Visualization of endometriotic lesions during laparoscopy has not been found to be reliable in confirming the diagnosis. Only 49% of lesions appearing positive for endometriosis were confirmed to be histologically positive^19^. IC is diagnosed with hydrodistension at cystoscopy and visualization of grade 2–3 glomerulations, or Hunner's lesions, or both. These diagnostic methods are invasive and expensive, require anesthesia and training, and involve a recovery period and the patient missing work. Moreover, they both involve potential risks, including infection, bladder rupture, vascular, and organ injury.

As IC and endometriosis have the same symptoms, the patient is likely to attribute menstrual worsening of symptoms to gynecological causes of pain and therefore seeks gynecological care^31^. Pain is usually the symptom that patients find most troublesome. They may disregard any other symptom including urinary symptoms as insignificant^31,32^. Patients may also fail to disclose any dyspareunia because of discomfort associated with its discussion. Gynecologic practice does not typically focus on urinary symptoms, and the diagnosis of IC is made by a gynecologist in only 8% of cases^33^. Approximately 75% of patients followed up with gynecological services for CPP have urinary symptoms, which often go unrecognized^12^. By contrast, urologic teaching does not typically focus on pelvic pain, menstrual exacerbations, or dyspareunia^3^. Cheng et al. reported that women generally present to endometriosis and pelvic pain units with the chief complaints of pain and dysmenorrhea. However, 93% of these women were found to have urinary symptoms^10^. Cystoscopic features of IC including Hunner's lesions are not easily recognized and require an experienced urogynecologist^10^. In addition, no presenting symptoms were found to correlate with cystoscopic findings of granulations^10^. Multiple studies have established that >80% of patients with CPP have positive PST, signifying that pain in these patients was either caused by IC alone or gynecological conditions in conjunction with IC^31^. Endometriosis is found in 15%–60% of patients who were asymptomatic undergoing laparoscopy for other indications^3,10,11^. Therefore, endometriotic nodules, if present, do not necessarily cause CPP. An alternate or concurrent diagnosis should be sought out, especially in inadequate pain control with treatment of endometriosis^3,10^. It is difficult to ascertain that, when found laparoscopically, the pain is attributed to endometriosis. According to Hurd, to attribute CPP to endometriosis, three criteria should be met: (1) cyclical pelvic pain due to the hormonally responsive nature of endometriosis, (2) the diagnosis of endometriosis should be made surgically and not based on the patient's symptoms, and (3) medical and surgical treatment should provide prolonged improvement in pain. Endometriosis may be asymptomatic, even if diagnosed in women with CPP^34^. IC should be considered in all women complaining of CPP. If undiagnosed, IC can cause unsuccessful medical and surgical therapy^14^.

The criteria set for diagnosis of IC by the National Institute of Diabetes and Digestive and Kidney Diseases (NIDDK) are stern. NIDDK published criteria for IC in 1988^35^. These criteria were formulated for research purposes and were not created to be used as a definition for IC^36^. The NIDDK criteria are based on expert opinion rather than on clinical evidence. Moreover, 60% of patients with IC would be missed by these stringent criteria^16,12,14,33^. IC has been interpreted differently in various centers and regions. Studies of the prevalence of IC engage widely variable methods of diagnosing and defining IC, which has caused prevalence rates to vary significantly from 1% to 11%^36,37^. No objective marker for IC has been identified for use in a diagnostic protocol^38^. It is difficult to ascertain the true prevalence of IC, which is changing with the evolving nomenclature. In addition, widely variable methods of diagnosing IC engaged by different researchers and centers have yielded highly varied prevalence^38^. The Rand Corporation Interstitial Cystitis Epidemiology (RICE) study was conducted to estimate the national prevalence of IC in American women. The study found that a single definition for PBS that has high sensitivity and specificity to distinguish PBS from other conditions has not been established. This may be due to the overlap of symptoms between IC/PBS and other conditions, including endometriosis, vulvodynia, and OAB. The sensitivity of the RICE high-sensitivity definition was 91%, and the specificity was low at 42%. However, the RICE high-specificity definition has a sensitivity of 56% and a specificity of 79%^38^. No highly sensitive and specific definition for IC was established^38^. Finally, many studies have been performed before the introduction of BPS nomenclature. Upon review of these studies, all IC cases were considered to have BPS, and the terms were used interchangeably.

## Conclusion

The rates of concurrent endometriosis and IC reported by different studies vary widely. However, all studies agree that the prevalence of the two concurrent conditions is high. The relationship between the two conditions is poorly understood, as the pathogenesis behind each condition is not known. Multiple theories, including that both conditions are caused by mast cell degranulation or neurogenic upregulation with viscerovisceral hyperalgesia, have been proposed but not yet proven. The coexistence of IC and endometriosis is frequently overlooked. The results of multiple studies have supported that this association is more likely to exist in patients with CPP than making the diagnosis of endometriosis or IC alone. However, it is difficult, if not impossible, to ascertain which of the two conditions is the major source of pain. Simultaneous assessment with cystoscopy and laparoscopy and treatment for both conditions are crucial, especially in patients who continue to experience symptoms despite previous treatment. The presence of endometriosis does not exclude IC as a cause of CPP. CPP may be caused by either or both of these conditions, raising the need to look beyond the conventional diagnosis of endometriosis in treating patients with CPP. This review emphasizes the importance of an interdisciplinary approach involving both urologists and gynecologists to avoid delayed or missed diagnosis as well as prevent unnecessary surgical interventions. Early referral of patients with CPP by clinicians for multidisciplinary care is paramount. In addition, clinician focus should consider the bladder as a possible generator of pain early in the workup.

Future research of the outcome and patient response is required when standardized diagnostic protocols are employed to investigate for both endometriosis and IC concurrently. This will produce a validated diagnostic approach to help the clinician in identifying patients with these potential diagnoses, ascertain a relevant history of symptoms, investigate other potential differential diagnoses, and rapidly test for IC and endometriosis. Less stringent criteria for diagnosis that are applicable in clinical practice are required. Homogenizing definitions and diagnostic criteria will eliminate differences between clinical practice and research.

## Figures and Tables

**Table 1:** Prevalence of non-cyclical chronic pelvic pain as cited by the World Health Organization systematic review determining the prevalence and geographic rates of chronic pelvic pain^2^.

USA	15%

UK	24%

India	5.2%

Pakistan	8.8%

Thailand	43.2%

